# Application of Micro/Nanoporous Fluoropolymers with Reduced Bioadhesion in Digital Microfluidics

**DOI:** 10.3390/nano12132201

**Published:** 2022-06-27

**Authors:** Andreas Goralczyk, Sagar Bhagwat, Fadoua Mayoussi, Niloofar Nekoonam, Kai Sachsenheimer, Peilong Hou, Frederik Kotz-Helmer, Dorothea Helmer, Bastian E. Rapp

**Affiliations:** 1 Laboratory of Process Technology, NeptunLab, Department of Microsystem Engineering (IMTEK), University of Freiburg, 79110 Freiburg im Breisgau, Germany; andreas.goralczyk@neptunlab.org (A.G.); sagar.bhagwat@neptunlab.org (S.B.); fadoua.mayoussi@neptunlab.org (F.M.); niloofar.nekoonam@neptunlab.org (N.N.); kai.sa@t-online.de (K.S.); peilong.hou@neptunlab.org (P.H.); frederik.kotz-helmer@neptunlab.org (F.K.-H.); bastian.rapp@neptunlab.org (B.E.R.); 2 Freiburg Materials Research Center (FMF), University of Freiburg, 79104 Freiburg im Breisgau, Germany; 3 Freiburg Center of Interactive Materials and Bioinspired Technologies (FIT), University of Freiburg, 79110 Freiburg im Breisgau, Germany

**Keywords:** superhydrophobic, digital microfluidics, micro/nanoporous, low bio-adhesion, EWOD, DMF

## Abstract

Digital microfluidics (DMF) is a versatile platform for conducting a variety of biological and chemical assays. The most commonly used set-up for the actuation of microliter droplets is electrowetting on dielectric (EWOD), where the liquid is moved by an electrostatic force on a dielectric layer. Superhydrophobic materials are promising materials for dielectric layers, especially since the minimum contact between droplet and surface is key for low adhesion of biomolecules, as it causes droplet pinning and cross contamination. However, superhydrophobic surfaces show limitations, such as full wetting transition between Cassie and Wenzel under applied voltage, expensive and complex fabrication and difficult integration into already existing devices. Here we present Fluoropor, a superhydrophobic fluorinated polymer foam with pores on the micro/nanoscale as a dielectric layer in DMF. Fluoropor shows stable wetting properties with no significant changes in the wetting behavior, or full wetting transition, until potentials of 400 V. Furthermore, Fluoropor shows low attachment of biomolecules to the surface upon droplet movement. Due to its simple fabrication process, its resistance to adhesion of biomolecules and the fact it is capable of being integrated and exchanged as thin films into commercial DMF devices, Fluoropor is a promising material for wide application in DMF.

## 1. Introduction

Digital microfluidics (DMF) is a versatile subfield of microfluidics and offers the advantage of operating multiple individual microdroplets simultaneously without the need for channels, valves or pumps, facilitating complex processes as a programmable sequence of multiple steps [1,2]. Recently, three-dimensional (3D) DMF devices have also been developed, allowing fluid transportation in all three spatial dimensions [3]. This potentiates various fields of application for DMF, such as immunoassays [4], real-time polymerize chain reaction (PCR) test for pathogens [5], mass spectrometry [6,7], cell culture and analysis [8], DNA ligation [9], plasma separation of blood [10] and, recently, COVID-19 testing [11]. The working principle utilized for droplet actuation in DMF is electrowetting on dielectric (EWOD), where, upon application of an electrostatic potential, a force acts on a liquid droplet manipulating its wetting behavior [12,13]. The basic EWOD setup consists of electrodes that are covered with a dielectric layer, which functions as an insulator, preventing electrolysis of the liquid, and as a hydrophobic layer, to reduce the liquid’s adhesive forces towards the solid surface, so maintaining a high mobility of the droplet [14]. The properties of the dielectric material are crucial for the performance of EWOD. The wettability of the surfaces plays a major role in the performance of the DMF devices, as it characterizes the adhesion between the liquid drop and the solid surface which needs to be overcome for droplet actuation. Therefore, the top coatings in EWOD are required to be hydrophobic materials, i.e., materials possessing static water contact angles (CAs) above 90°. DMF setups for droplet actuation can further be divided into open setups, where the droplet is on top of the dielectric [15,16,17,18,19,20], and closed setups, where a second hydrophobized layer covers the droplet from the top [21,22,23,24,25,26]. Open setups have the advantage of simple fabrication [27] and offer less surface area for biofouling. Most open systems consist of a thicker insulating material at the bottom and on top a thin hydrophobic coating, usually consisting of a low surface energy fluoropolymer [15,19,20,28].

An interesting class of materials for EWOD are superhydrophobic surfaces with static water CA larger than 150° and low roll-off angles (ROs) below 10° [29]. On such superhydrophobic surfaces, the droplet sits on top of the asperities of the surface structure and entraps a thin air-layer underneath, thus maintaining high mobility of the droplet in the so-called Cassie-Baxter (CB) wetting state [30]. It was shown that structured superhydrophobic surfaces exhibit a higher droplet movement speed [31] and higher cleaning efficiency than unstructured hydrophobic surfaces for different (bio-)particles with EWOD, demonstrating that it is advantageous to use these structured surfaces in DMF [32]. Various surface structures on the nanoscale [32,33,34,35,36,37,38,39,40,41], the microscale [42,43,44,45] and on both nanoscale and microscale [46,47] were examined in EWOD. These surfaces consisted of an insulating layer and a repellent low surface energy one. Materials used for insulation include silicon dioxide [33,35,37,44,47], photoresists, such as, for example, SU-8 [42] and zirconium oxide [39,45], while commonly used hydrophobic materials are fluoropolymers, such as C_4_F_8_ [33,35,37,44,47], Teflon [39,42], Cytop^®^ [32], or alkyl-/fluorosilanes [32,38,45] and carbon [36]. There are also systems that consist of one single layer that simultaneously serves as an insulating and repellent layer, examples of which include coatings with carbon nanotubes [34], graphene [46] and polypyrrole [48]. 

One major drawback hindering the wide application of superhydrophobic surfaces in DMF is the partial or full wetting transition that occurs upon application of a potential to the electrodes. Here, the droplet transits from the superhydrophobic CB state, to the so-called Wenzel-state, where the liquid touches the surface, fills the cavities of the surface topography and can further fill the structure by capillary forces. The latter state manifests itself in strong pinning behavior of the droplet to the surface, making these droplets immobile. There have been some approaches to transit back from the Wenzel to the CB state, such as heating of the surface [35], electrolysis of the impaled water [49], application of pressure from the backside of a superhydrophobic surface [50], vibration of the surface [51] or oscillation of the droplet with alternating modulated potentials [52]. Yet, the possible successful application of these strategies in DMF devices is limited. due to the requirement of more complex setups (heating structures, gas supply channels, additional electrolysis electrodes, vibration actuators), or their limited reversibility. Other drawbacks in the use of superhydrophobic materials are the expensive and complex fabrication processes for thin layers, often requiring cleanroom techniques, and the fragile nature of the superhydrophobic surface, which poses a problem, especially for the open setup of DMF. Finally, many applications involve biomolecules, such as peptides or DNA, which adhere to the dielectric layer surface causing changes in wetting behavior with droplet pinning as well as cross contamination [1,53]. Therefore, a superhydrophobic material which shows good inherent resistance towards wetting transitions, can be fabricated in a simple, fast and scalable manner, shows low adhesion of biomolecules and is insensitive to abrasion is highly sought-after. Moreover, as electrode fabrication is costly, a system with exchangeable dielectric layers would be favorable to reuse the electrodes.

Here we demonstrate a superhydrophobic, bulk micro/nanoporous fluoropolymer foam, “Fluoropor”, which we previously reported [54,55,56,57] as a promising dielectric material for EWOD and application in DMF. Fluoropor is easy to fabricate and, due to its bulk porous structure, insensitive to abrasion, as the removal of one layer of material exposes an equally structured layer of material. We examined the wetting behavior of Fluoropor under electrowetting and found that until 400 V of applied potential no significant changes in the wetting behavior occurred, maintaining the liquid droplet in mobile state. Aqueous droplets with CY-3™ labeled Streptavidin and an oligonucleotide were actuated ten times over the Fluoropor surface and showed no adhesion to it, which was examined by fluorescence microscopy. Moreover, we demonstrated droplet actuation on Fluoropor surfaces integrated in a commercial DMF device and assessed its performance by conducting the assay reaction of methylene blue and ascorbic acid. In summary, its stable wetting behavior, its resistance to adhesion of biomolecules and the fact that Fluoropor can be easily integrated and exchanged on already existing DMF devices make this material promising for wide application in DMF.

## 2. Materials and Methods

### 2.1. Materials

Fluorolink MD700 (MD700) was obtained from Acota (Oswestry, UK). The porogens 1*H*,1*H*,2*H*,2*H*-perfluorooctanol (13FOOl) and cyclohexanol (>99.0%) was purchased from Apollo Scientific (Stockport, UK) and Merck (Darmstadt, Germany), respectively. Acetone (>99.5%, synthesis grade), ethanol (>99.5%, denatured), hydrochloric acid (37% fuming, technical) and phosphate buffered saline (PBS) tablets were supplied by Carl Roth (Karlsruhe, Germany). *L*-ascorbic acid (>98%), 2,2-dimethoxy-2-phenylacetophenone (DMPAP), diphenyl(2,4,6-trimethylbenzoyl) phosphine oxide (TPO), methylene blue (certified by biological stain commission), customized Cy-3 labeled oligonucleotides (sequence: 5′-Cy3-AAA CGA CGC AGG AAA AAA AA-3′) and Streptavidin-Cy3™ from *Streptomyces avidinii* in buffered aqueous solution were purchased from Sigma-Aldrich (Germany). Tinuvin 384-2 was kindly provided by BASF (Ludwigshafen, Germany). Solvent free 2-component epoxy resin adhesive was obtained from UHU, Germany. Water, as well as nitrogen gas, was used from the in-house supply.

### 2.2. Methods

The wetting behavior was characterized using an OCA 15EC CA goniometer (DataPhysics Instruments, Filderstadt, Germany) and evaluated with SCA20 software. The static CA, roll-off angle (ROA), receding and advancing CA were measured for 5 µL size water droplets and recorded with 200 frames s^−1^. For the ROA experiments a tilting speed of 1.24 ° s^−1^ was set and receding and advancing CA were evaluated from the last frame before the droplet rolled off the surface. For the droplet shape evaluation by the software the ellipsoidal fitting method of the software was used, as it showed the best overlapping of computed droplet shape and actual droplet shape. For advancing and receding CA measurements, a tangential fitting method was utilized for droplet shape evaluation.

The micro/nanostructure of Fluoropor was characterized by scanning electron microscopy (SEM) utilizing a Quanta 250 FEG device (FEI Inc., Hillsboro, OR, USA) with 5 kV accelerating potential. The sample was prepared on an SEM-sample and sputtered with a circa 25 nm thick gold-palladium layer.

UV-VIS measurements were conducted to estimate the optical transparency utilizing an Evolution 201 UV-VIS spectrometer (Thermo Scientific, Karlsruhe, Germany).

The potential for the electrowetting experiments, as well as for the droplet actuation, was applied by a high voltage power supply (10/10B-HS, Trek Inc., Lockport, NY, USA) which was regulated over a DSO-X-2014A function generator (Agilent Technologies, Santa Clara, CA, USA). All applied potentials were DC voltage.

### 2.3. Experiments on the Wetting Behavior under EWOD

#### 2.3.1. Surface Preparation

**Figure 1:**
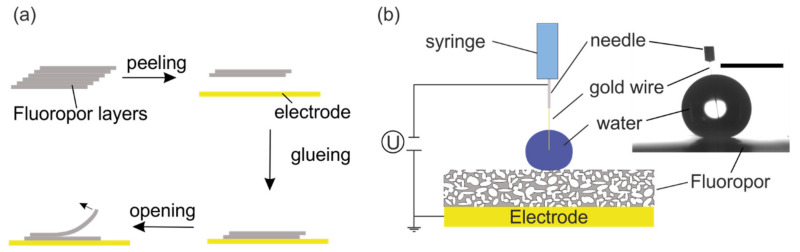
Fabrication process and measurement setup for the wetting behavior experiments: (**a**) Fabrication process of Fluoropor surfaces for wetting behavior experiments: From the 3D-printed stack of individual Fluoropor (grey) layers, a double layer (fabricated according to our previous work [56]) was peeled off and glued on a steel panel, which functioned as an electrode (yellow). After the adhesive cured, the protective top layer of the Fluoropor double layer was removed to open the target surface for the wetting experiment. (**b**) The setup of the wetting transition experiment consisted of the steel electrode, which was connected to the mass of the power supply, the porous Fluoropor coating on top of it and the water dispersing unit with the metal needle connected to the phase of the power supply. A thin gold wire inside the syringe facilitated electrical connection to the water droplet. Right: Actual image from the CA goniometer showing the droplet, which was connected over the thin gold wire to the syringe. Scale bar: 2 mm.

To achieve superhydrophobic micro/nanoporous surfaces, we used the previously reported fluoropolymer foam Fluoropor [54,55,56]. In general, amorphous fluorinated polymers are known to be good electric insulators [58] and lend themselves for the production of dielectric layers for DMF. Fluoropor is produced in a simple procedure, mixing fluorinated methacrylates with a porogen and emulsifier. During the polymerization, the molecular weight of the growing polymer increases and a phase separation occurs which causes the formation of a porous network. The combination of low surface energy monomers with a micro/nanoporous structure result in a superhydrophobic material. Moreover, Fluoropor can retain its superhydrophobicity, even if the surface is abraded, because abrasion reveals a new layer of identical pore structures, due to the bulk porosity of the material. Fluoropor surfaces that were used to examine the wetting behavior were fabricated by 3D SLA printing as a staircase design, according to the procedure we have reported recently [56,59]. Briefly, a 3D-printable resin was prepared, which consisted of 50 wt% MD700 monomer with 35 wt% of 13FOOl (emulsifying agent), 15 wt% cyclohexanol (porogen), blended with 0.5 wt% of TPO (photoinitiator) and 0.6 wt% of Tinuvin 384-2 (absorber). The staircase design was printed on an Asiga Pico 2 SLA printer (Asiga, Alexandria, NSW, Australia) with a light intensity of 88 W m^−2^ utilizing the same parameters as reported previously [56,59]. The printed stacks were washed in isopropanol overnight and dried at ambient conditions for 16 h, followed by drying in a vacuum furnace at 100 °C and 50 mbar for 1 h. The washing and drying procedure was required for removal of the porogens from the polymeric network and to obtain a superhydrophobic micro-/nanostructured polymer foam. A double-layer (two layers connected together) of Fluoropor was peeled off the stacked layer design and glued with a solvent free 2-component epoxy resin adhesive on stainless steel sheets (10 × 10 cm). After the adhesive was cured, the top-layer of the glued double-layer was peeled from the bottom layer to give a final Fluoropor layer of 131 ± 16 µm thickness (see Figure 1a).

#### 2.3.2. Electrowetting Experiments

The prepared Fluoropor surfaces on the steel panels were mounted on the CA goniometer. The mass of the power supply was connected to the steel panel and the phase linked to the syringe of the dispersing unit. A thin 25 µm thick gold wire (Heraeus, Germany) was guided through the syringe in such a manner that approximately 1 mm of it protruded from the capillary of the syringe (see Figure 1b). The purpose of the wire was to connect the dispersed droplet to the power supply, as utilizing the syringe directly as the connector would alter the drop shape, thereby more strongly influencing the measurement results. To determine the electrowetting behavior the following measurement protocol was established:

1. A 5 µL droplet was deposited on the Fluoropor surface and the syringe was moved up so only the thin gold wire was still in contact with the droplet. The CA was measured (CA_0_).
2. The potential was turned on ad-hoc with the target value and the CA was measured (CA_on_).
3. The potential was turned off and after 30 s the CA was measured (CA_off_).
4. The gold wire was gently removed from the droplet by moving the dispersion unit up. Then the ROA and the advancing and receding CA (ARCA) were measured upon tilting of the stage. To determine ROA, videos were recorded with 200 frames s^−1^ and the last frame before the drop started rolling was utilized to evaluate the advancing and receding CA (ARCA).

This protocol was conducted for each measuring point across a potential span from 50 and 500 V at 50 V increments. For measurements with potentials between 500 and 800 V, the potential was increased in 100 V increments to observe a stronger change of the CA. For each potential the measurement protocol was repeated three times with a fresh droplet on a fresh spot on the Fluoropor coating to avoid measuring on an already wetted area. In addition, the measurements were conducted on five different Fluoropor surfaces giving, in total, 15 data points for each potential.

### 2.4. Fluoropor Film Preparation for Droplet Actuation Experiments

Thin films of Fluoropor were fabricated by infusion of Hybriwell™ sealing chambers (75 × 25 × 0.15 mm, Grace Bio-Labs, Bend, OR, USA) which were stuck to a polycarbonate foil with liquid Fluoropor solution. The Fluoropor solution was prepared using 50 wt% MD700 with 35 wt% 13FOOl and 15 wt% cyclohexanol blended with 1 wt% of DMPAP with regard to the monomer dissolved in acetone (1 mg DMPAP in 1 µL acetone). The Fluoropor films were cured for 5 min utilizing a Hellas UV exposure unit with an intensity of 110 W m^−2^ (Bungard, Windeck, Germany), washed in acetone for one day and dried in a vacuum furnace (VacuTherm VT6025 from Thermo Scientific, Karlsruhe, Germany) at 100 °C and 30 mbar for 1 h. Finally, the films were fixed by vacuum on a Büchner funnel and opened by abrasion with sandpaper (grid 2000). Excessive polymer particles were removed from the surface by wiping them off with a glove. During the drying procedure the films shrank by circa 50% and abrasion further reduced the resulting thickness to circa 50 µm.

### 2.5. Chemical Assay

**Scheme 1:**
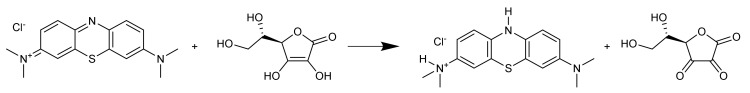
Chemical reaction of Methylene blue with ascorbic acid to colorless leuco form.

The reaction of methylene blue (MB) with ascorbic acid (Asc) in aqueous media was performed as a showcase for the application of Fluoropor in DMF (reaction see Scheme 1). For this, two stock solutions were prepared: The first one was a 0.28 M aqueous solution of Asc and the second one a 2.5 mM aqueous solution of MB with 0.88 M hydrochloric acid. The reaction was performed on an opened Fluoropor film mounted on the commercial DMF device OpenDrop V4 (GaudiLabs, Luzern, Swiss) by sticky tape. A 20 µL droplet of each solution was placed on the Fluoropor surface and then moved with 300 V DC potential.

### 2.6. Adhesion of Biomolecules

Adhesion to the surface was investigated by probing the surface with aqueous solutions of biomolecules. The adhesion of biomolecules to the superhydrophobic surface was investigated, on the one hand, for proteins with streptavidin and, on the other hand, with a customized oligonucleotide with 20 bases. A 20 µL droplet of each solution was moved ten times across a designated spot on the Fluoropor film which was mounted on the DMF device with 300 V DC potential. Both biomolecules contained a Cy-3 fluorescence label to detect their adhesion onto the surface by fluorescence microscopy. The measurements were performed on a fluorescence microscope (DMi8, Leica, Wetzlar, Germany) with Cy-3 filter and 200 ms exposure time.

## 3. Results and Discussion

### 3.1. Wetting Behavior on Fluoropor under EWOD

**Figure 2:**
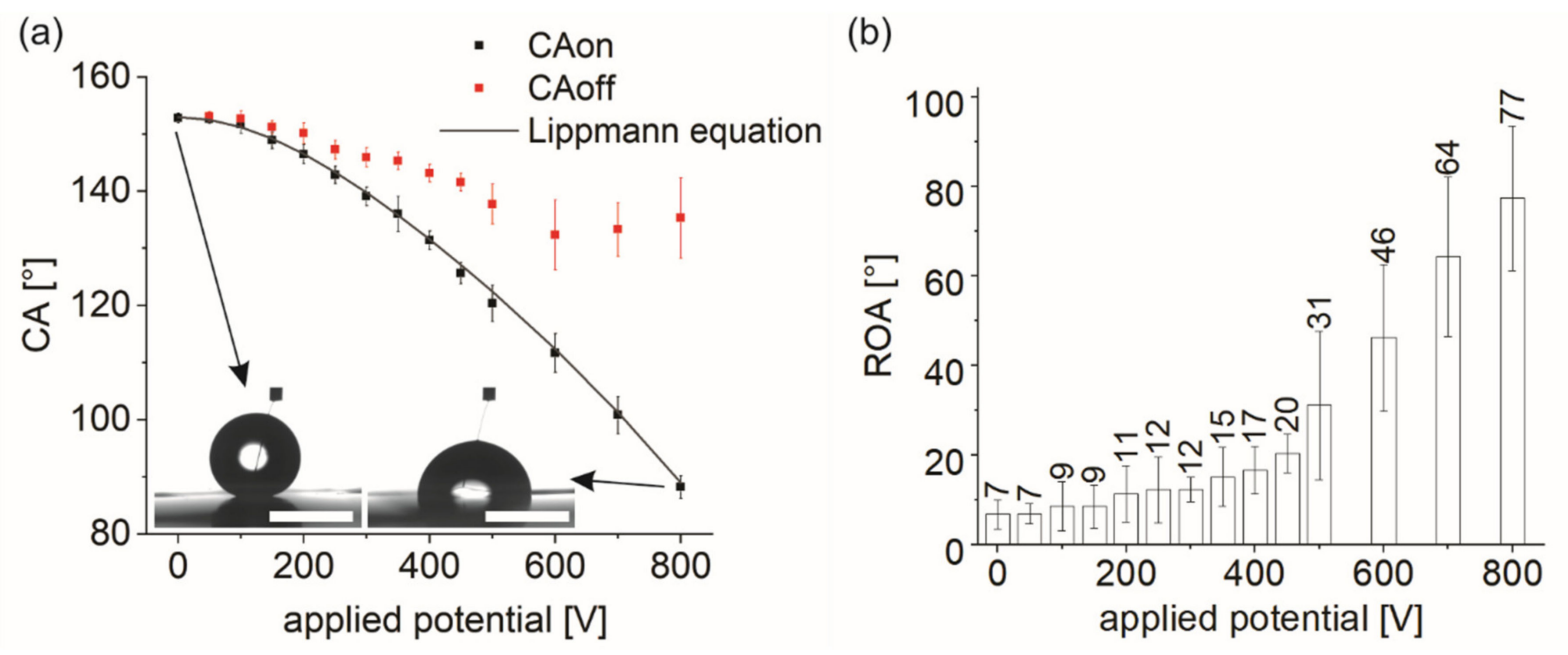
CA and ROA of water on Fluoropor (131 ± 16 µm) in dependence of the applied potential. (**a**) The CA with applied potential (CA_on_ !) decreased progressively with the increase of the applied potential. The analytical predication (black line) based on the Lippmann equation showed a good fit to the measured values. Two inset images show the actual water droplet shape at 0 V and 800 V applied potential. Scale bars: 2 mm. After the measurement, the potential was turned off and the contact angle was measured again (CA_off_ !). The contact angle increased for all measurements but never decreased below 133 ± 6°. This indicated a wetting transition to a stable wetting state in which the droplet partly imbibed the porous structure above 400 V. (**b**) After measuring CA_off_, ROA was measured showing an increase in ROA with increasing potentials. This indicated that the droplet rolling off was inhibited by the intrusion of the liquid into the material and, therefore, a wetting transition from CB to a Wenzel state.

**Figure A1:**
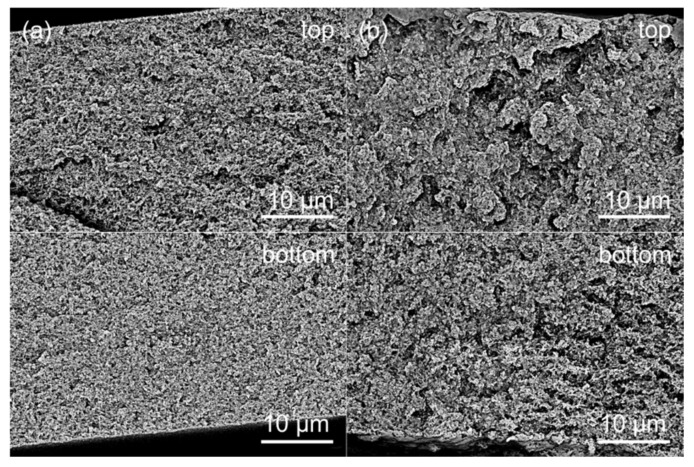
Cross-sectional micro-graphical images of Fluoropor with different thickness, showing the porous structure throughout the bulk material: (**a**) Fluoropor film of 75 µm and (**b**) 150 µm thickness (sections from the top and bottom of the material were chosen representatively).

Fluoropor is a superhydrophobic fluoropolymer foam with a porous structure on the micro/nanoscale throughout the bulk material which is independent of the thickness of the material (see Figure A1): The porosity was visible in the micrographical images throughout all of the bulk material for Fluoropor of different thicknesses. Thin layers of Fluoropor (131 ± 16 µm) were attached to stainless steel plates to examine the wetting behavior under EWOD. First, water droplets of 5 µL were placed on the Fluoropor surfaces and their initial CA (CA_0_) and ROA were measured: CA_0_ was 153 ± 1° and the ROA was measured to be 7 ± 3°. These values matched with the values for Fluoropor surface (see values for CA_0_ in Table A1) and served as proof that the surface was pristine. Next, a potential was applied to the droplet. It was observed that the CA decreased with increase in the applied potential (CA_on_) (see Figure 2a): CA_on_ changed from an initial 153 ± 1° with no applied potential to 88 ± 2° at 800 V. The higher the applied potential, the higher was the electrostatic force acting on the droplet and the lower was CA_on_. This correlation of the CA and the applied potential was investigated firstly by Gabriel Lippmann, who proposed the following equation [12]:

(1) $$\cos\;\theta =\cos\theta_{0}+\frac{\epsilon_{r}\cdot \epsilon_{0}\;\cdot U^{2}}{2\;d\;\cdot \gamma_{LV}}$$

where $\theta_{0}$ is the CA without applied potential, $\epsilon_{r}$ is the dielectric constant and d the thickness of the dielectric material, U is the applied potential, $\epsilon_{0}$ is the electric permeability in vacuum and $\gamma_{LV}$ is the surface tension of the utilized liquid. The CA with applied potential θ, was calculated for potentials between 0 and 800 V and compared to the measured CA_on_ in Figure 2a. For the calculation $\theta_{0}$ was set to 153°, d was 131 µm, $\epsilon_{0}$ is 8.854 × 10^−12^ A s V^−1^ m^−1^ and $\gamma_{LV}$ was 72.8 mN m^−1^. The dielectric constant $\epsilon_{r}$ was calculated by inserting the CA_on_ values for 800 V and solving the equation. A higher value for the dielectric constant is beneficial in DMF as it represents a higher permeation of the electric field through the material, resulting in lower potential required for droplet movement [14,60]. For our Fluoropor material, based on a perfluorinated dimethacrylate, the value was calculated to be 3.10, which is significantly higher than the values for regular fluoropolymers, such as PTFE with 2.01 and more in a range of materials, such as parylene C [60,61]. Therefore, Fluoropor was found here to be advantageous over other regularly used fluoropolymers, such as PTFE. Utilizing all constant values in the equation, the theoretical CA was calculated and inserted in Figure 2a which showed very good accordance with the measured values.

**Figure 3:**
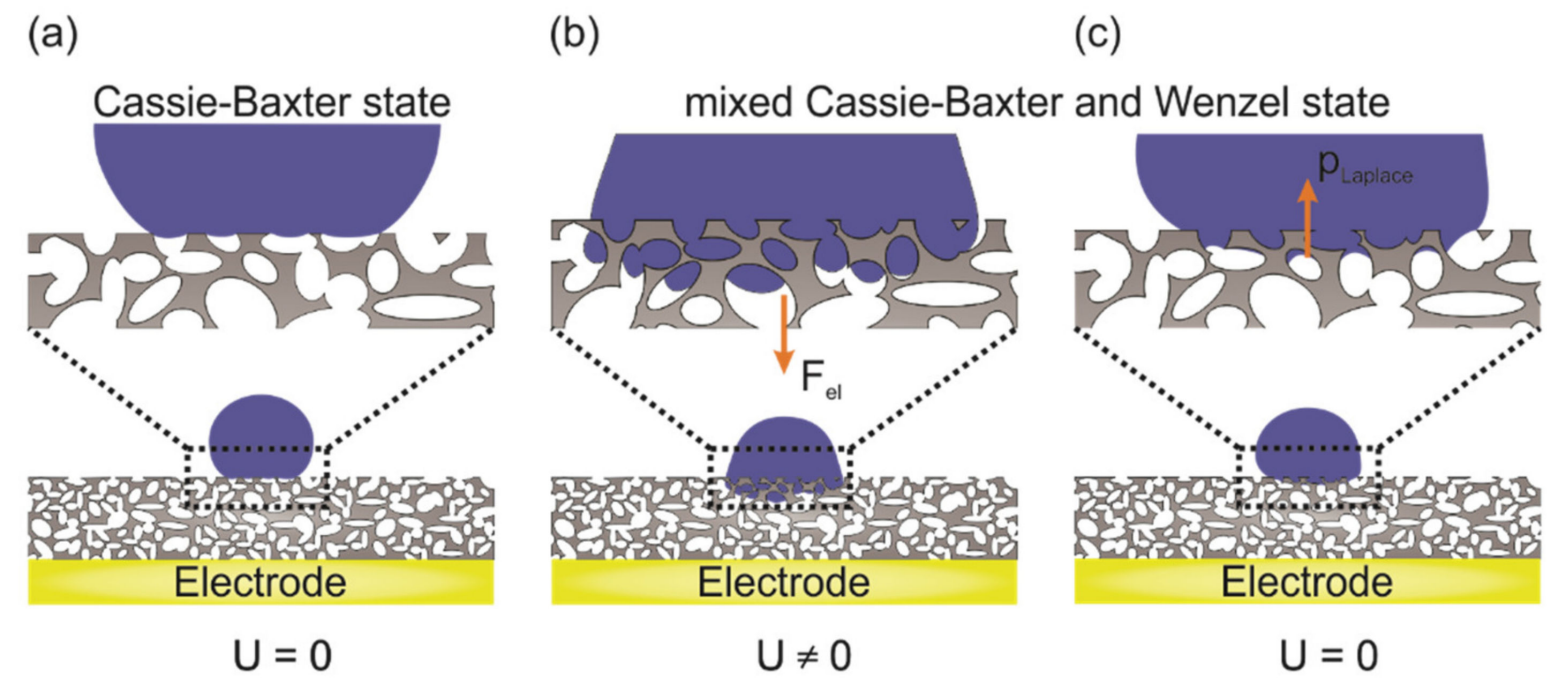
Schematic wetting states on a porous superhydrophobic surface, e.g., Fluoropor before, while and after application of a potential U: (**a**) In its initial wetting state the droplet sits on top of the asperities of the porous surface in the CB state. (**b**) Upon application of a potential, a downward pointing electrostatic force (F_el_) evolves and drags the droplet into the pore network, causing a decrease of the CA. (**c**) When the potential is turned off again the droplet is pushed out of the pores by the Laplace pressure (p_Laplace_) resulting in a decrease of the CA. However, the droplet cannot return into the initial wetting state.

After the applied potential was turned off, the CA was measured again to give CA_off_. The droplet receded from the Fluoropor coating and an increase in CA was observed (see CA_off_ in Figure 2a). Yet, it was observed that the droplet could not return to its initial state of manifesting due to the fact that CA_off_ was lower than the initial CA_0_ (see Table A1). It was observed that CA_off_ decreased from an initial 153 ± 1° with increasing voltage but never decreased below 133 ± 6° (see Figure 2a). Additionally, not only CA_off_, but also the ROA of the droplet was evaluated to determine whether the adhesion of the droplet to the material increased during electrowetting. The data showed a slow increase of the ROA with increase of the applied potential, with only slight changes occurring until a voltage of 400 V (see Figure 2b). Above 400 V the ROA increased drastically. This behavior could be explained by the forces acting on the droplet, and similar wetting transition behavior with electrowetting has been previously reported on different structured surfaces [45,62,63,64]: Initially the droplet was in the superhydrophobic CB state. In this state the droplet was sitting on top of the asperities, keeping a thin air layer inside the cavities of the superficial pores (see Figure 3a). This state was characterized by a high CA and low ROA, as observed. For a stable CB state, the forces acting on the droplet must be in equilibrium resulting neither in spreading nor de-wetting of the droplet on the surface. When a potential was applied to the droplet, a CA decrease was observed, while the droplet spread on the Fluoropor coating. Upon application of an electric potential to the droplet an additional force, i.e., the electrostatic force, evolved, pulling the droplet into the pores (Figure 3b). Entering of the liquid into the pores caused an increase in the Laplace pressure, as the curvature of the liquid’s meniscus entering the pores increased. The droplet moves downwards until the electrostatic force is balanced by the increasing Laplace pressure [62,65]. It was shown that with higher electrowetting potential the liquid entered the surface structure, and thereby caused an increase in the repelling pressure, which was calculated based on the Laplace-Young pressure [45,64]. Especially for porous surfaces the repelling pressure increases drastically when the liquid enters the channel interconnecting the cavities, due to the small channel diameter and increased curvature of the meniscus [45]. In our experiment by applying a potential, the droplet transitioned from the initial CB state to a mixed CB and Wenzel state, where local fully wetted pores (Wenzel state) coexisted with local non-wetted or partly wetted pores [62]. This mixed CB-Wenzel wetting state has already been outlined for the case of the liquid entering into the porous structure and wetting the superficial pores but not fully penetrating the whole bulk of the material [45]. When the potential was turned off the droplet receded from the Fluoropor coating and the CA increased. Turning off the potential also meant that the resulting downwards pulling electrostatic force was being removed. Then the Laplace pressure (p_Laplace_) pushed the droplet out of the pores until the system was in thermodynamic equilibrium again (see Figure 3c). The Laplace pressure was approximated for our material, with a median pore diameter of 161 nm and a water CA on the flat surface of 114°, to be 0.36 MPa (calculation described in Appendix A). The values for the median pore diameter and the water CA were based on findings from our previous work [66]. Furthermore, it was reported in the literature that the CA increased and the liquid de-wetted the previously penetrated surface when the potential was turned off [62]. For this to occur, it is required that the Wenzel wetting state is less stable than the CB state [67].

**Figure 4:**
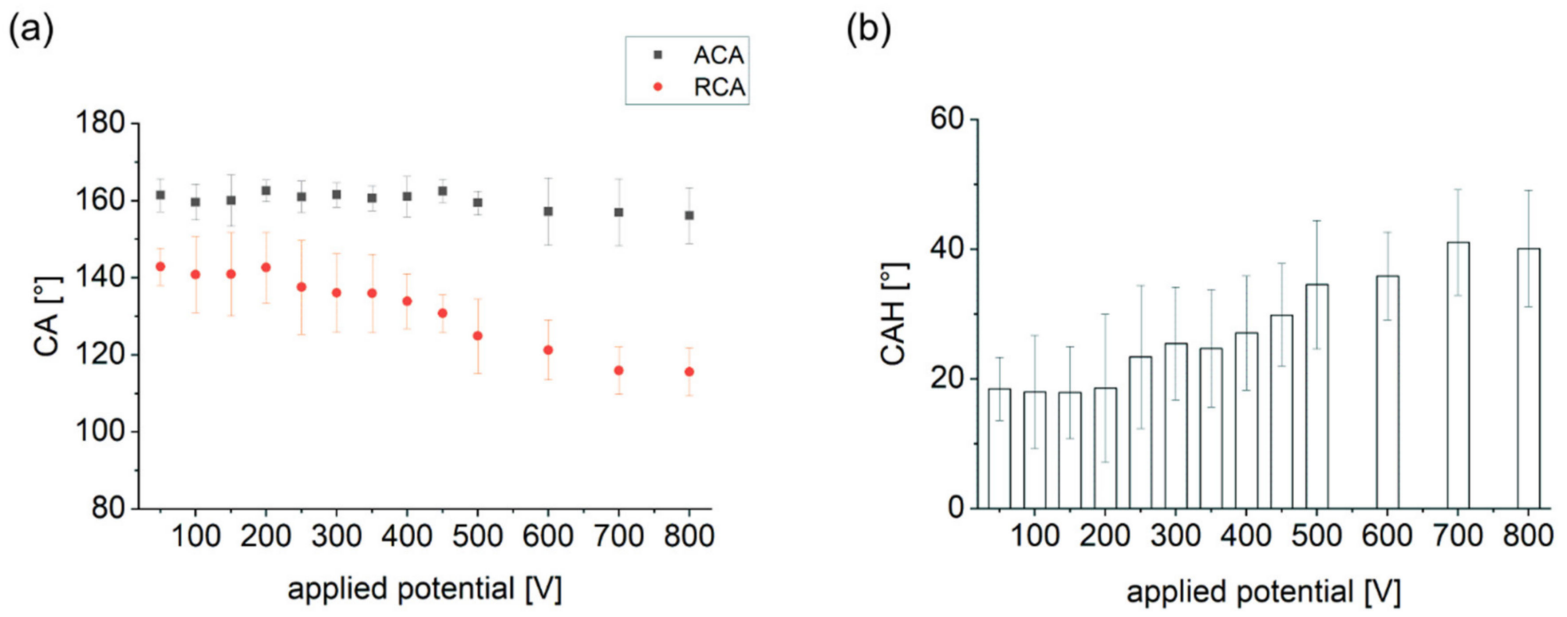
Measured advancing and receding CA (ARCA) and subsequent calculated CA hysteresis (CAH) in dependence of the applied voltage. (**a**) ARCA measurement revealed that with the increase of the potential the advancing CA (ACA, !) was stable while the receding CA (RCA) decreased. (**b**) This resulted in an increase of the CAH.

Besides CA and ROA measurements, advancing and receding CA measurements were also conducted as they provide crucial information on the droplet mobility in electrowetting [68]. With the increase of the applied potential the receding CA (RCA) decreased from 143° to 116°, while the advancing CA (ACA) remained rather stable at 160° (see Figure 4a). The difference of both these CAs is known as the CA hysteresis (CAH), which increased from 19° to 40° with increase of the applied potential (see Figure 4b). The measured and calculated values are summarized in Table A2. The RCA is related to the pinning behavior of a material and will increase the stronger the pinning forces of the droplet to the surface are. Our findings confirmed the results from the ROA measurements where, till 400 V applied potential, no significant changes occurred, meaning the wetting behavior did not change significantly in this region. However, above 400 V decrease of the RCA indicates the partial wetting transition of the droplet. In brief, this measurement also showed the ability of Fluoropor to keep the droplet in a mobile state even at a high voltage.

In brief, with applied potential the wetting transition from initial CB state to a mixed CB and Wenzel state occurred, which could be partly reversed once the electrostatic force was removed. Yet this reversibility decreased when the electrostatic forces were higher, which could be explained by the liquid entering deeper into the pore network [45]. However, no full transition from CB to Wenzel, where the droplet cannot roll-off anymore, was observed. In fact, till 400 V applied potential, the changes of the wetting behavior by means of ROA and RCA were not significant, showing that the droplet could be maintained in the highly mobile wetting state. This ability of Fluoropor might be attributed to the special pore network and is beneficial for droplet actuation in DMF.

### 3.2. Droplet Actuation on Fluoropor Films

**Figure 5:**
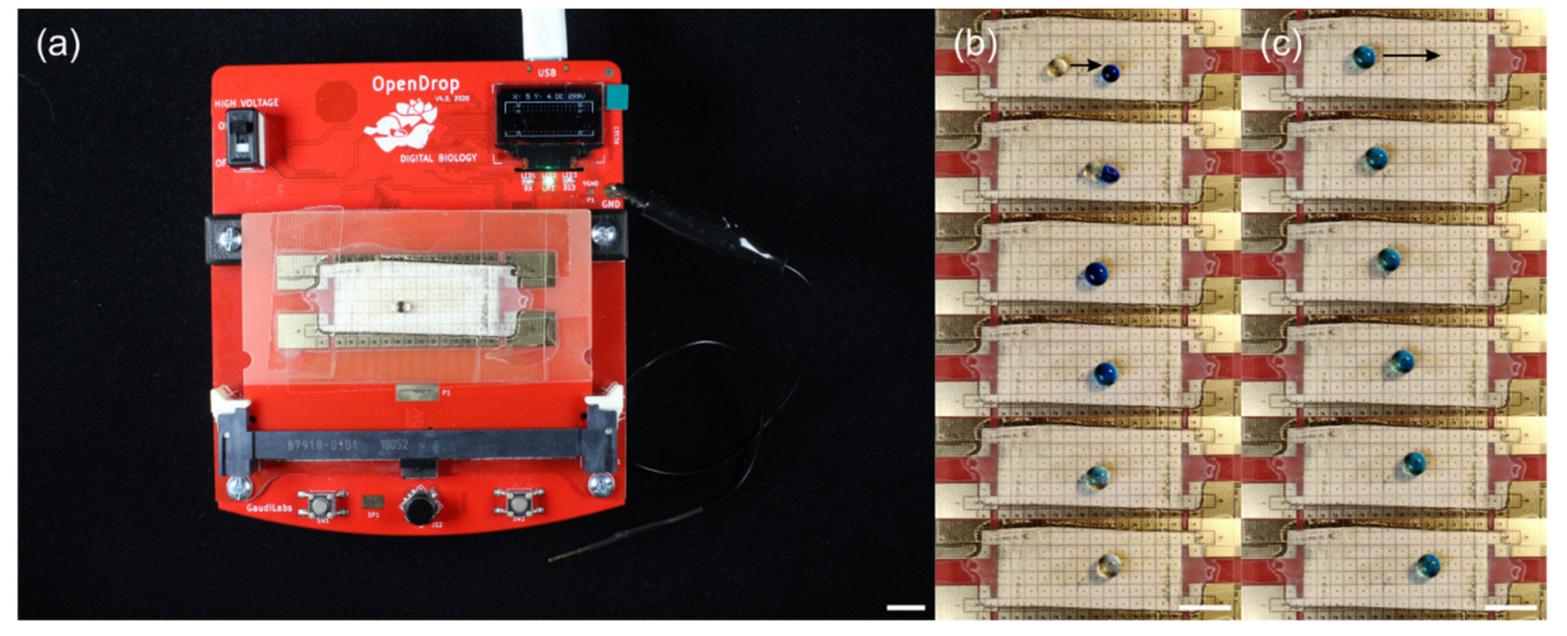
Droplet actuation on the commercial DMF OpenDrop device for performing a chemical assay. (**a**) DMF device OpenDrop with Fluoropor film mounted on the electrode array with a 20 µL sized water droplet on top. Scale bar: 10 mm. (**b**) A chemical assay on the Fluoropor-covered electrodes by mixing of an Asc droplet (transparent) with a MB droplet (blue) resulted in a gradual discoloration of the MB after mixing, due to the reaction of the educts to the colorless product. (**c**) Droplet movement on Fluoropor film by electrowetting of a 30 µL sized droplet with MB and Asc from left to right as indicated by the arrow. Scale bar: 10 mm.

**Figure A2:**
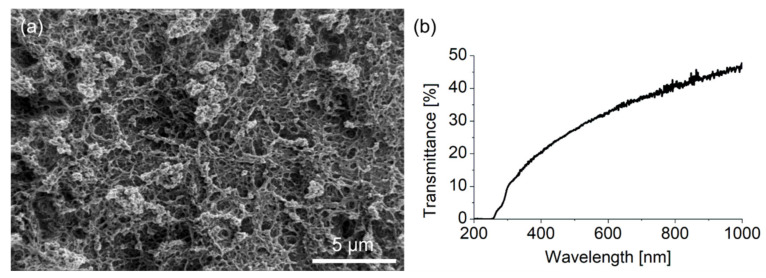
Analysis of structure and transmission of Fluoropor films: (**a**) Micro-graphical image of a Fluoropor film used on the DMF device for droplet actuation showing the micro/nanoporous structure of Fluoropor. (**b**) UV-VIS transmission spectra of a 51 µm thick Fluoropor film showing moderate transparency for visible light.

We tested the ability of Fluoropor to keep droplets in a mobile state even at high potentials in a DMF experiment. Thin Fluoropor films (thickness 51 ± 4 µm, CA 159 ± 1°, ROA 4 ± 2°, see Figure A2a for an SEM view, see Figure A2b for UV/Vis transmittance) were used as dielectric layers on the commercial DMF device OpenDrop. The Fluoropor films were mounted on the electrode array of the DMF device by adhesive tape (see Figure 5a). In order to adhere the Fluoropor film tightly to the electrodes, a thin film of silicon oil was suspended on the electrode array prior to covering it. A chemical assay was performed on the DMF device, i.e., the reaction of MB and Asc in aqueous media. For this, first, a transparent 15 µL droplet of Asc was placed on top of a powered electrode on the DMF device followed by placing a blue 15 µL droplet of MB four electrodes away (see Figure 5b). Next the Asc droplet was moved with a potential of 300 V DC towards the MB droplet until both merged and the colors were mixed. The droplet was then moved over the electrode array while the color gradually disappeared until the droplet was transparent (see Movie S1). This loss of color is an inherent indicator of the reaction in which MB reacts with Asc to the colorless leuco form of MB, which manifested itself in the gradual discoloration of the aqueous droplet (see Scheme 1) [69]. This reaction was chosen as a showcase for it was easy to monitor the progress of the reaction visually when the droplet changed its color from blue to colorless. It was shown that the droplet possessed a high mobility on the DMF device and could be moved precisely over the Fluoropor surface without pinning (see Figure 5c). 

**Figure A3:**
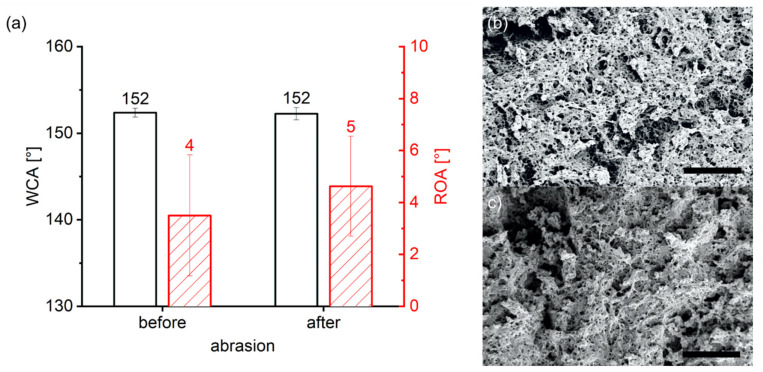
Robustness of Fluoropor films showing stable repellence and surface structure: (**a**) The WCA and ROA were measured with 5 µL water droplets on the Fluoropor surface before and after abrasion, showing that no significant change of the repellence upon abrasion. (**b**) Micro-graphical image of Fluoropor15 before and (**c**) after 100 abrasive cycles showing no significant change. Scale bar: 5 µm.

These results showed that Fluoropor could be used as a dielectric material for DMF and could be implemented into an already existing system of electrodes, hardware and software. Moreover, Fluoropor could be exchanged as the dielectric material while the DMF device could be reused with a fresh Fluoropor film. Most of the reported setups used for DMF involve electrodes that are directly coated with the dielectric layer which cannot then be separated from one another, thus, preventing their reuse. As electrode fabrication is expensive, the reuse of the electrodes would be favorable and we showed here that this was possible with Fluoropor [17]. Additionally, Fluoropor showed robustness to abrasive impact (see Figure A3): The CA did not change before (152 ± 1°) and after abrasion (152 ± 1°) with 100 g loaded sandpaper for 100 cycles. Additionally, the surface showed stable RO (before 4 ± 2°, after 5 ± 2°). This robustness was attributed to the special bulk-porous structure of Fluoropor [54].

### 3.3. Adhesion of Biomolecules

**Figure 6:**
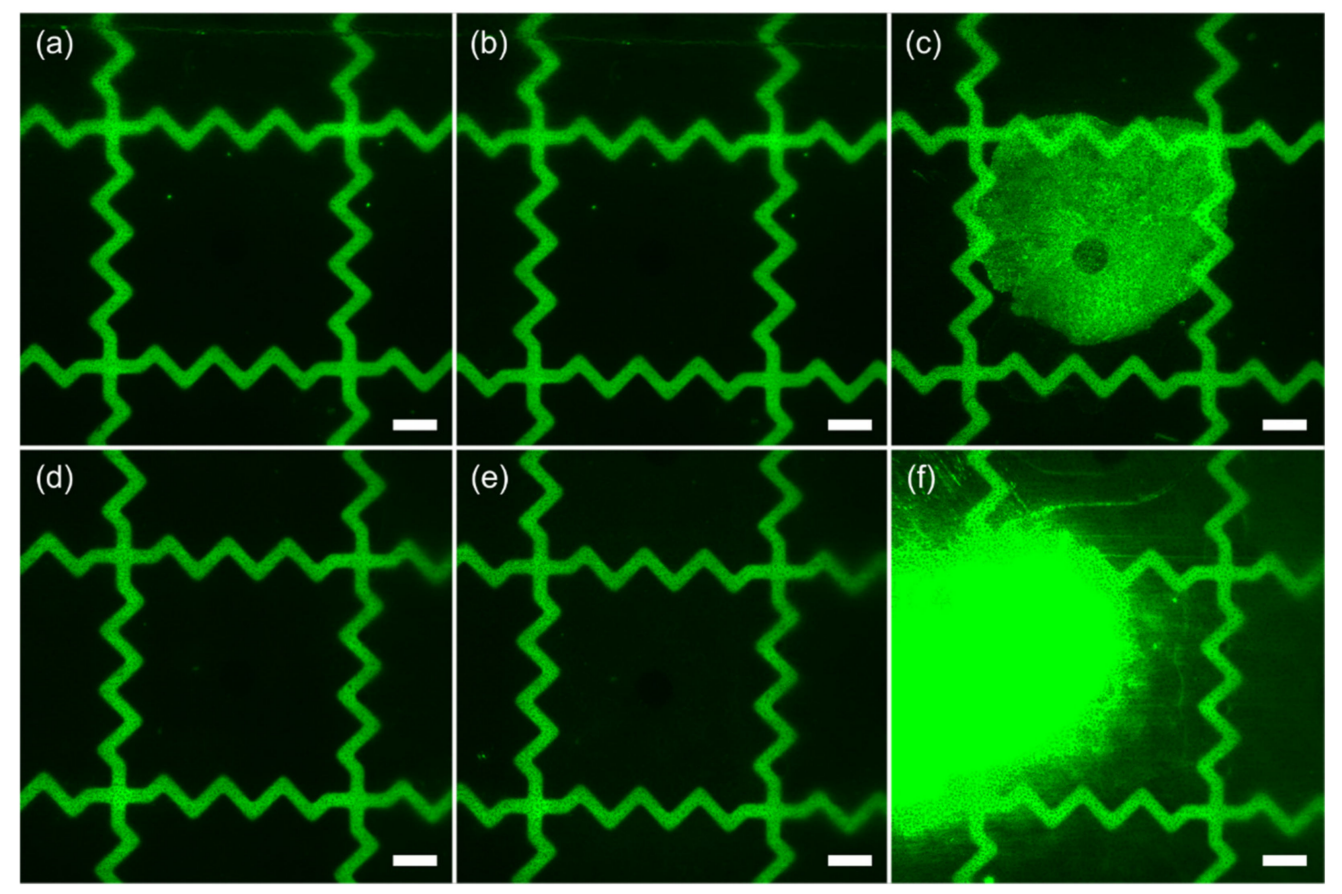
Fluoropor films mounted on the electrodes of the DMF device were examined for the adhesion of biomolecules after actuation by electrowetting by fluorescence microscopy. (**a**) Microscopic image of a Fluoropor film before actuation of a 20 µL droplet of aqueous biomolecule solution. (**b**) After a droplet of Streptavidin-Cy3 was moved ten times over the electrode no staining of the Fluoropor surface was observed. (**c**) For comparison the repellence was broken down with ethanol followed by placing a Streptavidin water droplet on the Fluoropor surface. A clear staining of the surface was observed. Analogously an aqueous solution containing an oligonucleotide was moved ten times over the surface. The Fluoropor surface showed no staining before (**d**) and after (**e**) droplet movement. (**f**) Again, the repellence was broken using ethanol with subsequent immersion of the oligonucleotide droplet resulting in detectable surface staining. As can be seen, Fluoropor does prevent adhesion of biomolecules, as long as the inherent repellence of the material is not prevented. Scale bars: 500 µm.

When it comes to biochemical assays one major drawback of dielectrics used in DMF is adhesion of biomolecules, such as proteins or DNA, to the surface leading to pinning and cross-contamination [1,53]. Therefore, it is necessary that dielectric materials show a low adhesion for these molecules for their application in biochemistry. To test our surfaces, we probed them with Streptavidin and an oligonucleotide in an aqueous solution to examine the adhesion of those biomolecules to the surface. Both biomolecules were labeled with a Cy3™ fluorescence dye to enable the evaluation of bio-adhesion by fluorescence microscopy: when a droplet containing biomolecules enters the surface, i.e., when the droplet is not in a superhydrophobic state, residual biomolecules cover the wetted surface and can be visualized by bright sports in fluorescence microscopy. Both solutions were in a superhydrophobic state on the Fluoropor film and were moved over the surface by electrowetting on the DMF device with 300 V DC. The droplet was moved ten times over the same spot, which was then analyzed for residual biomolecules. For the protein Streptavidin there was no difference found between the Fluoropor surface before and after probing (see Figure 6a,b), i.e., no bio-adhesion was detected. To obtain a reference image for the case when the surface was wetted, the repellent barrier was broken down by wetting with ethanol and then a droplet of Streptavidin was placed on this spot. A clear staining of the surface was observed for this case (see Figure 6c), showing bio-adhesion happened there. Analogous to the staining experiment with Streptavidin, the surface was also probed with an aqueous solution containing an oligonucleotide. Similarly, no staining was observed after probing of the surface with the oligonucleotide solution (see Figure 6d,e), i.e., no bio-adhesion was detectable. Analogously to the Streptavidin experiment, the surface was forcefully wetted by ethanol resulting in detectable staining (see Figure 6f) showing bio-adhesion. In conclusion, Streptavidin and the oligonucleotide did not adhere to the Fluoropor surface if the droplet was maintained in a superhydrophobic state and no bio-adhesion could be detected when droplets were moved by electrowetting over the surface.

## 4. Conclusions

In this work, we examined the potential application of Fluoropor, a micro/nanoporous fluoropolymer foam, in DMF. We studied the wetting behavior of Fluoropor with EWOD and found that a partial wetting transition from CB to a mixed CB and Wenzel state occurs, but no full wetting transition was observed, even at high potentials of 400 V. In this regime, no significant changes of ROA and RCA were detected. This showed that the droplet could be maintained in a mobile state without pinning to the surface throughout a large variety of potentials. To show the high mobility of the droplets, even at high potentials, a DMF experiment was conducted. Thin superhydrophobic Fluoropor films were fabricated with thicknesses of around 50 µm and integrated in a DMF device and aqueous droplets containing ascorbic acid and methylene blue could be moved easily over the Fluoropor surface. We examined whether bio-adhesion, which is a common limitation for surfaces in DMF, was an issue using aqueous droplets containing either proteins or DNA. After actuation of these droplets over Fluoropor, no detectable bio-adhesion to this surface was observed, making Fluoropor the material of choice for biochemical applications. In summary, Fluoropor was fabricated in an easy manner and showed good resistance to wetting transition with EWOD, no detectable bio-adhesion and was able to be integrated and exchanged on commercial DMF devices, which allowed the reuse of the electrodes. This makes Fluoropor a desirable material for DMF and is promising for a wide range of applications in future work, such as biochemical assays or analytical reactions.

## Data Availability

Data are contained within the article or Supplementary Material.

## Supplementary Materials

The following are available online at https://www.mdpi.com/article/10.3390/nano12132201/s1, Movie S1: Reaction of Methylene blue and ascorbic acid on the DMF device.

## Appendix A

The measured CAs before (CA_0_) while (CA_on_) and after (CA_off_) application of potential as well as the ROAs are summarized below:

**Table A1:** Measured CAs and ROAs from wetting behavior experiments.

Applied Potential [V]	CA_0_ [°]	CA_on_ [°]	CA_off_ [°]	ROA [°]
0	153 ± 3°	-	-	7 ± 3°
50	153 ± 1°	153 ± 1°	153 ± 1°	7 ± 2°
100	153 ± 1°	152 ± 2°	153 ± 2°	9 ± 6°
150	153 ± 1°	149 ± 2°	151 ± 1°	9 ± 5°
200	153 ± 2°	146 ± 2°	150 ± 2°	11 ± 6°
250	153 ± 1°	143 ± 2°	147 ± 2°	12 ± 7°
300	153 ± 1°	139 ± 2°	146 ± 2°	12 ± 3°
350	153 ± 1°	136 ± 3°	145 ± 2°	15 ± 7°
400	153 ± 1°	131 ± 2°	143 ± 2°	17 ± 5°
450	153 ± 1°	126 ± 2°	142 ± 2°	21 ± 4°
500	152 ± 1°	120 ± 3°	138 ± 4°	31 ± 17°
600	153 ± 1°	112 ± 3°	132 ± 6°	46 ± 16°
700	153 ± 2°	101 ± 3°	133 ± 5°	64 ± 18°
800	152 ± 1°	88 ± 2°	135 ± 7°	77 ± 16°

When the droplet is dragged by electrostatic forces into the material the Laplace pressure evolves as a counteracting force, pushing the liquid out of the pores. It can be approximated by the following equation

(A1) $$p_{Laplace}=\frac{-2\;\gamma \cos\left(\theta_{0}\right)}{d_{c}}$$

where γ is the surface tension of the liquid, which is for water 72.8 mN m^−1^, $\theta_{0}$ the WCA on the flat unstructured Fluoropor surface and $d_{c}$ the diameter of the capillary. From our previous work it was known that $\theta_{0}$ is 114° and the median pore diameter was 161 nm [66]. With these values the resulting Laplace pressure could be calculated as 0.36 MPa.

**Table A2:** Measured advancing (ACA) and receding (RCA) CAs and subsequent calculated CA hysteresis (CAH) in dependence of the applied potential.

Applied Potential [V]	ACA [°]	RCA [°]	CAH [°]
50	161 ± 4°	143 ± 5°	19 ± 5°
100	160 ± 5°	141 ± 10°	18 ± 9°
150	160 ± 7°	141 ± 11°	18 ± 7°
200	163 ± 3°	143 ± 10°	19 ± 11°
250	161 ± 4°	138 ± 12°	23 ± 11°
300	162 ± 3°	136 ± 10°	25 ± 9°
350	161 ± 3°	136 ± 10°	25 ± 9°
400	161 ± 5°	134 ± 7°	27 ± 9°
450	163 ± 3°	131 ± 5°	30 ± 8°
500	159 ± 3°	125 ± 10°	35 ± 10°
600	157 ± 9°	121 ± 8°	36 ± 7°
700	157 ± 9°	116 ± 6°	41 ± 8°
800	156 ± 7°	116 ± 6°	40 ± 9°

Robustness of Fluoropor films was examined on Fluoropor films that were glued to glass slides. The Fluoropor films were abraded with a 100 g loaded sandpaper (grid 2000) for 100 cycles by manual pulling over the surface. Repellence (WCA and ROA) was examined with 5 µL droplets before and after abrasion (see Figure A3a). Micro-graphical images of the surface before and after abrasion were made (Figure A3b,c). The results showed that the surface could sustain 100 abrasive cycles without change of its properties, showing good robustness.
